# A novel spheroid-based co-culture model mimics loss of keratinocyte differentiation, melanoma cell invasion, and drug-induced selection of ABCB5-expressing cells

**DOI:** 10.1186/s12885-019-5606-4

**Published:** 2019-04-29

**Authors:** Julia Klicks, Christoph Maßlo, Andreas Kluth, Rüdiger Rudolf, Mathias Hafner

**Affiliations:** 1 Institute of Molecular and Cell Biology, Mannheim University of Applied Sciences, Paul-Wittsack-Straße 10, 68163 Mannheim, Germany; 2 Institute of Medical Technology, Mannheim University of Applied Sciences and Medical Faculty Mannheim of Heidelberg University, Theodor-Kutzer-Ufer 1-3, 68167 Mannheim, Germany; 3 RHEACELL GmbH & Co. KG, Im Neuenheimer Feld 517, 69120 Heidelberg, Germany; 4 TICEBA GmbH, Im Neuenheimer Feld 517, 69120 Heidelberg, Germany

**Keywords:** 3D in vitro model, ABCB5, HaCaT, Melanoma, SK-MEL-28, Spheroid

## Abstract

**Background:**

Different 3D-cell culture approaches with varying degrees of complexity have been developed to serve as melanoma models for drug testing or mechanistic studies. While these 3D-culture initiatives are already often superior to classical 2D approaches, they are either composed of only melanoma cells or they are so complex that the behavior of individual cell types is hard to understand, and often they are difficult to establish and expensive.

**Methods:**

This study used low-attachment based generation of spheroids composed of up to three cell types. Characterization of cells and spheroids involved cryosectioning, immunofluorescence, FACS, and quantitative analyses. Statistical evaluation used one-way ANOVA with post-hoc Tukey test or Student’s t-test.

**Results:**

The tri-culture model allowed to track cellular behavior in a cell-type specific manner and recapitulated different characteristics of early melanoma stages. Cells arranged into a collagen-IV rich fibroblast core, a ring of keratinocytes, and groups of highly proliferating melanoma cells on the outside. Regularly, some melanoma cells were also found to invade the fibroblast core. In the absence of melanoma cells, the keratinocyte ring stratified into central basal-like and peripheral, more differentiated cells. Conversely, keratinocyte differentiation was clearly reduced upon addition of melanoma cells. Treatment with the cytostatic drug, docetaxel, restored keratinocyte differentiation and induced apoptosis of external melanoma cells. Remaining intact external melanoma cells showed a significantly increased amount of ABCB5-immunoreactivity.

**Conclusions:**

In the present work, a novel, simple spheroid-based melanoma tri-culture model composed of fibroblasts, keratinocytes, and melanoma cells was described. This model mimicked features observed in early melanoma stages, including loss of keratinocyte differentiation, melanoma cell invasion, and drug-induced increase of ABCB5 expression in external melanoma cells.

**Electronic supplementary material:**

The online version of this article (10.1186/s12885-019-5606-4) contains supplementary material, which is available to authorized users.

## Background

Melanoma is a malignancy that arises from uncontrolled proliferation and metastasis of neoplastic melanocytes. Normally, melanocytes are located in the most basal epidermal layer, attached to a collagen-IV rich basement membrane, which separates epidermal and dermal compartments of the skin [1, 2]. Malignant melanoma is the most lethal form of skin cancer [3]. Its incidence continues to increase each year and is currently responsible for more than 80% of deaths from skin cancer [4]. The majority of melanoma mutations are C > T transitions caused by ultraviolet light irradiation [5], which mostly affect the mitogen-activated protein kinase pathway [6–9]. When diagnosed in its early ‘non-tumorigenic’ stages, resection of the lesion results in very high survival rates [10]. In this period, which is also termed as radial growth phase [11], pigmented patches of skin (nevi) increase laterally in size and become palpable, but melanoma cells typically still reside within the epidermis and are not metastasis competent [12]. Nonetheless, already at this point, they affect cellular behavior in their local environment. For example, neoplastic melanocytes induce hyperproliferation and impair differentiation of keratinocytes [13]. Once further mutations have mediated metastasis competence, the lesion becomes ‘tumorigenic’ and enters the vertical growth phase, during which the morphology of nevi often switches from plaque to balloon-like [12]. Melanoma cells lead to breakdown of the basement membrane, massively invade the dermal and hypodermal compartments and metastasize to distant organs. Surgery is then no longer sufficient and the disease becomes much more challenging to treat [3, 14, 15]. Treatment options for late stage melanomas include kinase inhibitors and immunotherapies like the BRAF inhibitor vemurafenib and the anti-cytotoxic T-lymphocyte antigen-4 antibody ipilimumab [16–18]. However, monotherapy is unlikely to yield a long-term benefit due to multi-drug resistance and, therefore, combination therapies with different targeted and immunotherapies as well as standard chemotherapeutics are being evaluated [19–22]. In melanoma cells, ATP-binding cassette (ABC) transporters, in particular of type ABCB5, were found to mediate resistance to the chemotherapeutics doxorubicin and temozolomide [23, 24]. Although ABCB5 is present in several human tissues, it is highly abundant in melanocyte progenitors, melanoma cell lines, and melanoma biopsies [23, 25–28]. Furthermore, its expression correlates with tumor progression and metastasis competence [29].

For the reasons of simplicity, convenience, and cost, in vitro studies on melanoma are often performed in 2D-cell culture assays. However, gene expression is significantly different between 2D and 3D melanoma cultures, likely affecting the signaling exerted by and the sensitivity to drugs of melanoma cells [30]. Furthermore, the interactions between different cell types of tumor and stroma are difficult to model in 2D. Thus, to better mimic the in vivo situation, different 3D-cell culture approaches with several degrees of complexity have been developed, including spheroids, tumorospheres, human skin equivalents, and melanoma-on-chips assays [31] as well as xenografts of human melanoma spheroids in rodent recipients [32]. Such formats are very useful for basic and applied melanoma research, but the currently existing models are either composed of only melanoma cells or they are so complex that the behavior of individual cell types is difficult to understand, and often they are then hard to establish and expensive. In the present work, we describe a novel, simple spheroid-based melanoma model composed of fibroblasts, keratinocytes, and melanoma cells. It allows to track cellular behavior in a cell-type specific manner and recapitulates different characteristics of early melanoma stages. The different cell types arranged into a collagen-IV rich fibroblast core, a ring of keratinocytes, and groups of highly proliferating melanoma cells on the outside. Some melanoma cells were also regularly found to invade the fibroblast core. While in the absence of melanoma cells the keratinocyte ring stratified into central basal-like and peripheral, more differentiated cells, addition of melanoma cells clearly reduced keratinocyte differentiation. Treatment with the cytostatic drug, docetaxel, which has been primarily tested for combination therapy of melanoma [33–35], restored keratinocyte differentiation and ablated external melanoma cells. The few remaining external melanoma cells, however, showed a significantly increased amount of ABCB5-immunoreactivity.

## Methods

### Cell culture

The human fibroblast cell line CCD-1137Sk (ATCC® CRL-2703™) was cultured in Iscove’s Modified Dulbecco’s Medium (IMDM), with L-Glutamine, supplemented with 10% fetal bovine serum (Sigma), and 1% Penicillin Streptomycin (Capricorn). The human keratinocyte cell line HaCaT (CLS order no. 300493) and the human malignant melanoma cell line SK-MEL-28 (CLS order no. 300337) were cultured in Dulbecco’s Modified Eagle Medium (DMEM) High Glucose (4.5 g/l), with L-Glutamine, with Sodium Pyruvate (Capricorn) supplemented with 10% fetal bovine serum, and 1% Penicillin Streptomycin. Cells were maintained at 37 °C in 5% CO_2_. Cell lines were obtained in 2016 and repeatedly authenticated by phenotypic analysis, including expression of collagen IV for CCD-1137Sk, establishment of a CK10/CK14 gradient in 3D for HaCaT, and high proliferation rate for SK-MEL-28. Mycoplasma tests using the MycoAlert™ Mycoplasma Detection Kit (Lonza) were routinely performed to ensure mycoplasma-free cell cultures.

### 3D spheroid cultures and docetaxel treatment

Spheroids were prepared using 96- and 384-well cell repellent plates (Greiner). For mono-culture spheroids, fibroblasts (10,000 cells/well) and HaCaT cells (20,000 cells/well) were seeded. For skin bi-cultures, 10,000 cells of each, fibroblasts and keratinocytes, were used per well, and HaCaT cells were added three days after formation of the fibroblast core. Mono- and bi-cultures were cultured for  seven days. For tri-culture spheroids, fibroblasts (10,000 cells/well) were seeded. After three days, HaCaT (10,000 cells/well) and SK-MEL-28 cells (2500 cells/well) were added simultaneously. To distinguish between the different cell lines, CellTracker Fluorescent Probes (Life Technologies) were used. Before adding cells to the 3D co-culture, HaCaT cells were labeled with CellTracker Red CMPTX dye (Life Technologies, C34552) and SK-MEL-28 cells were labeled with CellTracker Green CMFDA (Life Technologies, C2925), each for a time period of 45 min according to the CellTracker manuals. Another two days later, tri-culture spheroids were treated with 100 nM docetaxel or 0.01 ‰ of DMSO as control for 15, 24, 48, and 72 h, respectively. Stock solutions (10 mM) of docetaxel (Sigma) were prepared in dimethylsulfoxide (DMSO). After treatments, spheroids were normally fixed and immunostained as described below. For some experiments, spheroids were transferred to 3D agarose molds (Sigma, Z764051) on day five after seeding in cell repellent plates. Treatment with DMSO or 100 nM docetaxel for 72 h, as well as fixation and cryosectioning were then carried out in the molds.

### Immunofluorescence

Immunostaining of spheroids used the following steps. Spheroids were collected in an Eppendorf tube, washed once with PBS (137 mM NaCl, 2.7 mM KCl, 10 mM Na_2_HPO_4_ × 2 H_2_O, 2 mM KH_2_PO_4_, pH 7.4), and fixed with 4% wt/vol paraformaldehyde in PBS at room temperature for 30 min. Then, spheroids were incubated overnight at 4 °C in 15% sucrose in PBS, followed by an incubation overnight at 4 °C in 25% sucrose (Roth, 4621.1) in PBS, before they were embedded in OCT (Leica Biosystems). A CM-1950 cryostat (Leica Biosystems) was used for preparing 10-μm-thick sections. 3D molds were washed once with PBS and fixed with 4% wt/vol paraformaldehyde in PBS at room temperature for 30 min. Next, molds were embedded in OCT and cut with a cryostat into 20-μm-thick sections. All sections were permeabilized with 0.1% Triton X-100 (Roth, 3051.4) in PBS, blocked with 3% BSA (Roth, 8076.3) in PBS, and stained with rabbit anti-Ki67 (Merck, AB9260), rabbit anti-cleaved caspase 3 (CAS3)(Cell Signaling, 9661), rabbit anti-cytokeratin 10 (CK10)(Thermo Fisher Scientific, PA5–32459), rabbit anti-collagen IV (Rockland, 600–401-106S), mouse anti-cytokeratin 14 (CK14)(Merck, MAB3232), or mouse anti-ABCB5 (3C2-1D12 [29]; and Thermo Fisher Scientific, MA5–17026) antibodies, followed by goat anti-rabbit Alexa Fluor 647 (Invitrogen, A21246), goat anti-mouse Alexa Fluor 555 (Invitrogen, A21424), or donkey anti-mouse Alexa Fluor 647 (Invitrogen, A31571) secondary antibody labeling. Nuclei were stained with Dapi (Sigma, 10,236,276,001). Finally, sections were washed with PBS and mounted with Mowiol (Roth, 0713.2) for confocal microscopy (SP8, Leica).

### Statistical analysis

Images were composed using Adobe Illustrator (Adobe Systems Software) and ImageJ. All numeric data were handled using Microsoft Excel 2013 and were subsequently incorporated into the Adobe Illustrator composite. Quantitative analysis of Ki67-, CAS3-, and CK10-positive cells was performed using ImageJ. Graphs are presented as mean ± SEM and statistically analyzed using one-way ANOVA with post-hoc Tukey HSD Calculator or Student t-test. *P*-values are indicated as * < 0.05, ** < 0.01.

## Results

### Characterization of spheroid keratinocyte and fibroblast mono- and bi-cultures

**Fig. 1:**
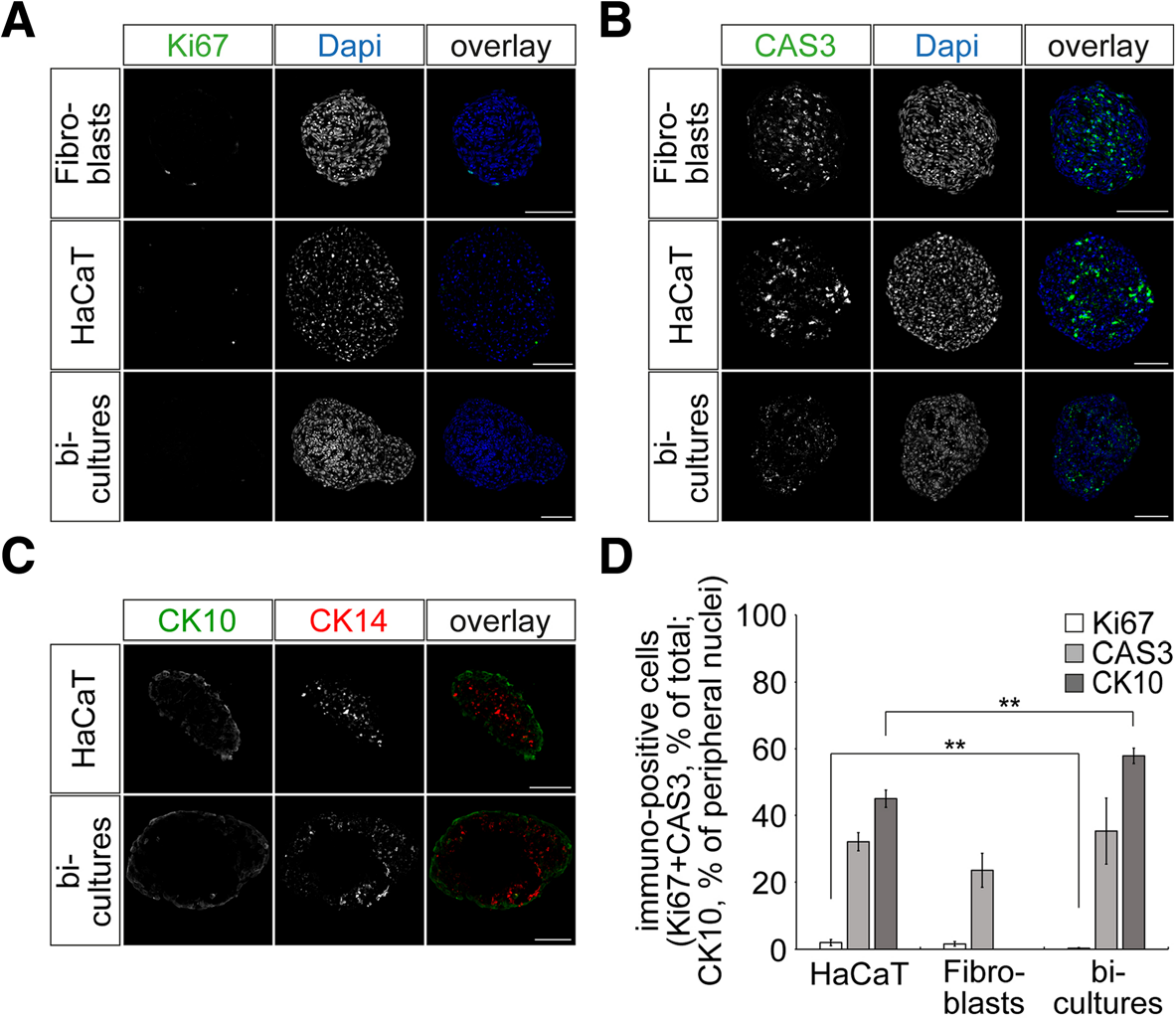
Proliferation, apoptosis, and differentiation of fibroblasts and HaCaT cells in mono- and bi-culture spheroids. Spheroids were cultured as mono- and bi-cultures for seven days, cryosectioned into 10-μm thick slices, and then stained for markers for cell proliferation (**a**, Ki67, green), apoptosis (**b**, CAS3, green), differentiated (**c**, CK10, green) and basal keratinocytes (**c**, CK14, red). In **a** and **b**, nuclei were stained with Dapi (blue). **a-c** Representative confocal images. Scale bars: 100 μm. **d** Quantification of Ki67- and CAS3-positive cells (percentage of total) and CK10-positive cells (percentage of peripheral nuclei). Given is mean ± SEM (n ≥ 3 independent experiments; ** *P* < 0.01)

To set up a simple, robust and multiplexable melanoma test system, we first tested the growth and differentiation behavior of major components of the stroma-like environment of melanoma, i.e. human keratinocytes and fibroblast cells, in 3D spheroids and also analyzed potential effects of co-culturing. Thus, both cell types were either cultured as mono- or co-cultures using a cell-repellent culturing system in 96-well format with a culture time of seven days. Then, spheroids of all types were cryosectioned into 10-μm thick slices and immunostained for the proliferation marker Ki67 or the apoptosis marker CAS3. Cytokeratins CK10 and CK14 were immunostained to detect more differentiated and basal keratinocytes, respectively. Nuclei were labeled with Dapi. Figure 1a-c show representative confocal sections of these samples as indicated. While proliferation was limited to few cells in the periphery of spheroids (Fig. 1a), apoptotic cells were found throughout the whole spheroid diameter (Fig. 1b). In both, mono-cultures and bi-cultures, HaCaT keratinocytes showed a clear stratification with basal-like CK14-positive and more differentiated CK10-positive cells in the center and on the periphery of the spheroids (Fig. 1c), respectively. In bi-cultures, fibroblasts formed a central core, while keratinocytes were located in a ring-like fashion around this fibroblast core. Quantitative analysis showed that co-culturing significantly reduced the number of proliferating and increased the amount of CK10-positive peripheral keratinocytes (Fig. 1d). These results suggest that the 3D spheroid skin model reflects some stratification and differentiation features of skin even without the use of a pH or Ca^2+^ gradient or an air-liquid interface to induce differentiation.

### Melanoma cells invade the fibroblast core and decrease keratinocyte differentiation in tri-cultures

**Fig. 2:**
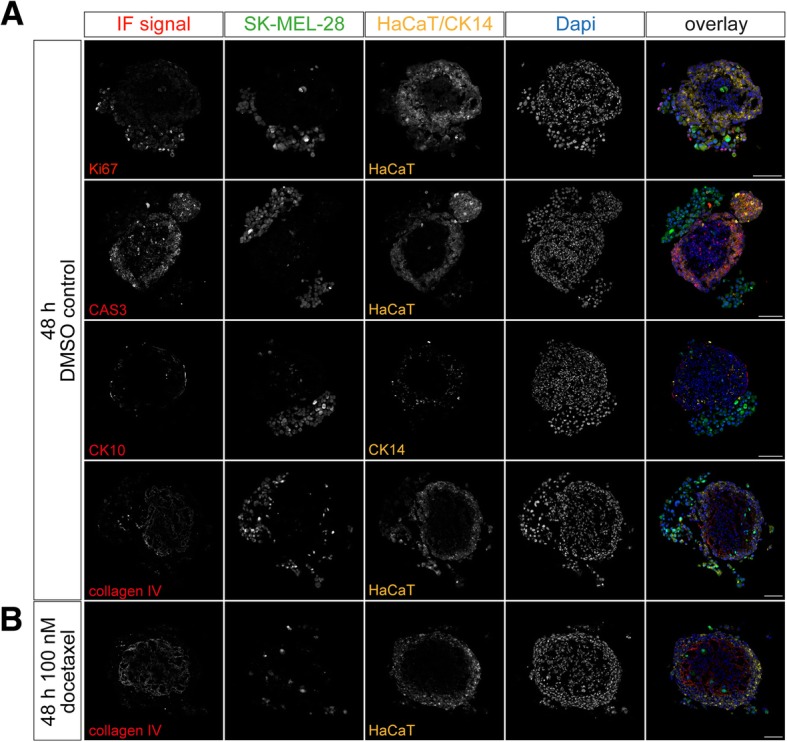
Characteristics of a melanoma tri-culture spheroid model. Tri-culture spheroids were made by 3D cultivation of CCD-1137Sk fibroblasts for three days, followed by simultaneous addition of HaCaT keratinocytes and SK-MEL-28 melanoma cells, and then further culturing for another four days. HaCaT and SK-MEL-28 cells were labeled with CellTrackerRed CMPTX and CellTrackerGreen CMFDA dyes, respectively. As indicated, spheroids were incubated on day five after seeding either with 0.01 ‰ of DMSO as control (**a**) or 100 nM docetaxel in DMSO (**b**) for 48 h, then cryosectioned into 10-μm-thick slices and stained for Ki67, CAS3, CK10, CK14, and the basal membrane marker collagen-IV. Nuclei were labeled with Dapi. Images show representative confocal sections through these samples. In overlay panels, all immunostainings except for CK14 are depicted in red, SK-MEL28 cells in green, HaCaT cells or CK14 in yellow, and nuclei in blue. Scale bars: 100 μm

After these initial characterizations, the spheroid skin model was complemented by the addition of SK-MEL-28 melanoma cells. Therefore, fibroblasts were seeded and cultivated in 3D. Three days later, HaCaT keratinocytes and SK-MEL-28 melanoma cells were added simultaneously. To distinguish between the different cell types, HaCaT and SK-MEL-28 cells were labeled with CellTrackers Red CMPTX and Green CMFDA, respectively. After another four days, tri-culture spheroids were harvested, cryosectioned into 10-μm thick slices and stained for Ki67, CAS3, CK10 and CK14, or the basement membrane marker collagen IV. As shown in Fig. 2a, fibroblasts remained in the central core of these tri-cultures, followed by a few layers of keratinocytes. While most melanoma cells were grouped in clusters of several dozens of cells on the shell of the cultures, a few melanoma cells were very regularly also found in the fibroblast core, but almost never in the keratinocyte layers. In the following, for simplicity, SK-MEL-28 cells in the outer rim of the tri-cultures will be termed ‘external’, those in the fibroblast core as ‘internal’ melanoma cells. A schematic representation of the tri-culture composition is depicted in Additional file 1: Figure S1A. The qualitative analysis further showed that numerous external melanoma cells were proliferating, while internal melanoma cells, keratinocytes, and fibroblasts were rarely doing so. Interestingly, CK10 expression as an indicator of keratinocyte differentiation was strongly reduced at the contact sites with melanoma cells.

### Docetaxel treatment affects external but not internal melanoma cells

To evaluate the tri-culture as a model system for testing drug candidates, we here chose to try a cytostatic, docetaxel, which is being explored in particular for combination treatments of malignant melanoma [33–35]. To find a useful concentration for in vitro tests, a dose-response curve was prepared. After their proper formation, tri-cultures were incubated for 48 h with different concentrations of docetaxel, i.e. 0 nM, 10 nM, 50 nM, 100 nM, 500 nM, and 1000 nM (Additional file 2: Figure S2). As exemplified in Fig. 2b, incubation with 100 nM of docetaxel strongly affected external melanoma cells and led to their nearly quantitative loss. Conversely, neither internal melanoma cells nor fibroblasts nor keratinocytes showed obvious defects. Quantification of remaining external melanoma cells after 48 h as a primary surrogate end point showed that already the lowest docetaxel concentrations slightly reduced external melanoma cells, but the effects were statistically significant in this setting only at concentrations ≥100 nM of docetaxel (Additional file 2: Figure S2). Therefore, in all following experiments this drug concentration was used.

### Docetaxel treatment of tri-culture spheroids decreases melanoma cell proliferation

**Fig. 3:**
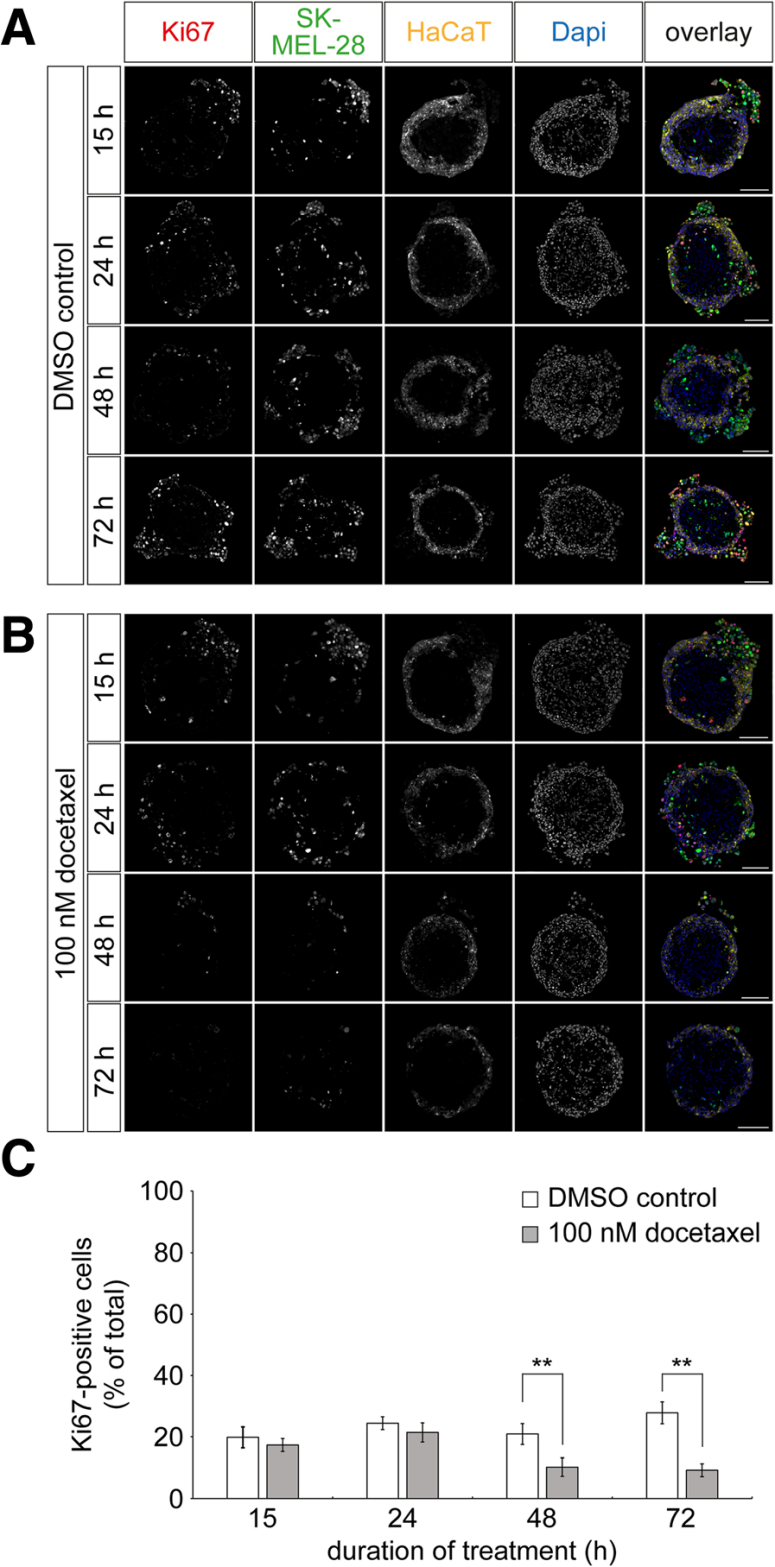
Docetaxel treatment reduces the amounts of proliferating cells in melanoma 3D tri-cultures. Tri-culture spheroids were produced by 3D cultivation of fibroblasts for three days, followed by simultaneous addition of keratinocytes and melanoma cells. HaCaT cells and SK-MEL-28 cells were labeled with CellTrackerRed CMPTX dye and CellTrackerGreen CMFDA, respectively. After another cultivation period of two days, tri-culture spheroids were treated with 0.01 ‰ of DMSO as control or 100 nM docetaxel for 15, 24, 48, and 72 h. Spheroids were cryosectioned into 10-μm thick slices and stained for Ki67. **a** and **b** Representative confocal images of control (**a**) and docetaxel-treated cultures (**b**). In overlay images, Ki67 immunostaining signals, SK-MEL-28, HaCaT, and nuclei are depicted in red, green, yellow, and blue, respectively. Scale bars: 100 μm. **c** Quantification of Ki67-positive cells. Graph displays the amounts of Ki67-positive cells as mean ± SEM (n ≥ 3 independent experiments; ** P < 0.01) in percent of the whole cell count per slice. For each experiment and time point, ≥ 3 spheroids were analyzed

To get an insight into the kinetics of docetaxel effects on the proliferation of the tri-culture spheroids, these were harvested after 15, 24, 48, and 72 h of treatment with 100 nM of docetaxel. Samples were cryosectioned and slices were stained for the proliferation marker Ki67. Figure 3a and b depict representative fields of view. DMSO controls showed a continuous increase in the number of external SK-MEL-28 cells over time (Fig. 3a, Table 1) and between 82.4% ± 2.4% (mean ± SEM, at 15 h) and 79.1% ± 3.2% (mean ± SEM, at 72 h) of those cells were proliferating. Conversely, docetaxel-treated spheroids led to increasing ablation of external melanoma cells (Fig. 3b, Table 1). Notably, survival of internal SK-MEL-28 cells in the fibroblast core was apparently not affected by docetaxel. Since external melanoma cells constituted the major source of proliferating cells in untreated tri-cultures, their selective loss upon docetaxel treatment reduced the fraction of Ki67-positive cells in the entire tri-culture from 20.9% ± 3.4% (mean ± SEM, *n* = 5 independent experiments, Fig. 3c) to 10.2% ± 3.0% (mean ± SEM, n = 5 independent experiments, Fig. 3c) after 48 h of treatment. 72 h after start of treatment, the difference was even higher with 9.2% ± 2.1% (mean ± SEM, *n* = 4 independent experiments, Fig. 3c) compared to 27.8% ± 3.6% (mean ± SEM, *n* = 3 independent experiments, Fig. 3c) in the presence and absence of docetaxel, respectively. In summary, these data demonstrate that docetaxel affects proliferating cells, which are in this model primarily external melanoma cells.

**Table 1:** Docetaxel progressively reduces the numbers of external SK-MEL-28. Tri-culture spheroids were harvested after 15, 24, 48, and 72 h of treatment with 100 nM of docetaxel or 0.01 ‰ of DMSO. HaCaT and SK-MEL-28 cells were labeled with CellTrackerRed CMPTX and CellTrackerGreen CMFDA dyes, respectively. Upon cryosectioning into 10-μm thick slices, labeling of nuclei with Dapi, and confocal imaging, external SK-MEL-28 cells were counted using ImageJ. Shown is mean ± SEM of the number of external SK-MEL-28 cells over time (*n* ≥ 3 independent experiments)

	DMSO control	100 nM docetaxel
15 h	80.4 ± 6.6	81.1 ± 4.0
24 h	77.6 ± 6.9	58.4 ± 6.2 **
48 h	142.9 ± 20.5	33.1 ± 3.1 **
72 h	135.2 ± 1.9	19.7 ± 3.2 **

### Docetaxel treatment does apparently not affect apoptosis in tri-culture spheroids

**Fig. 4:**
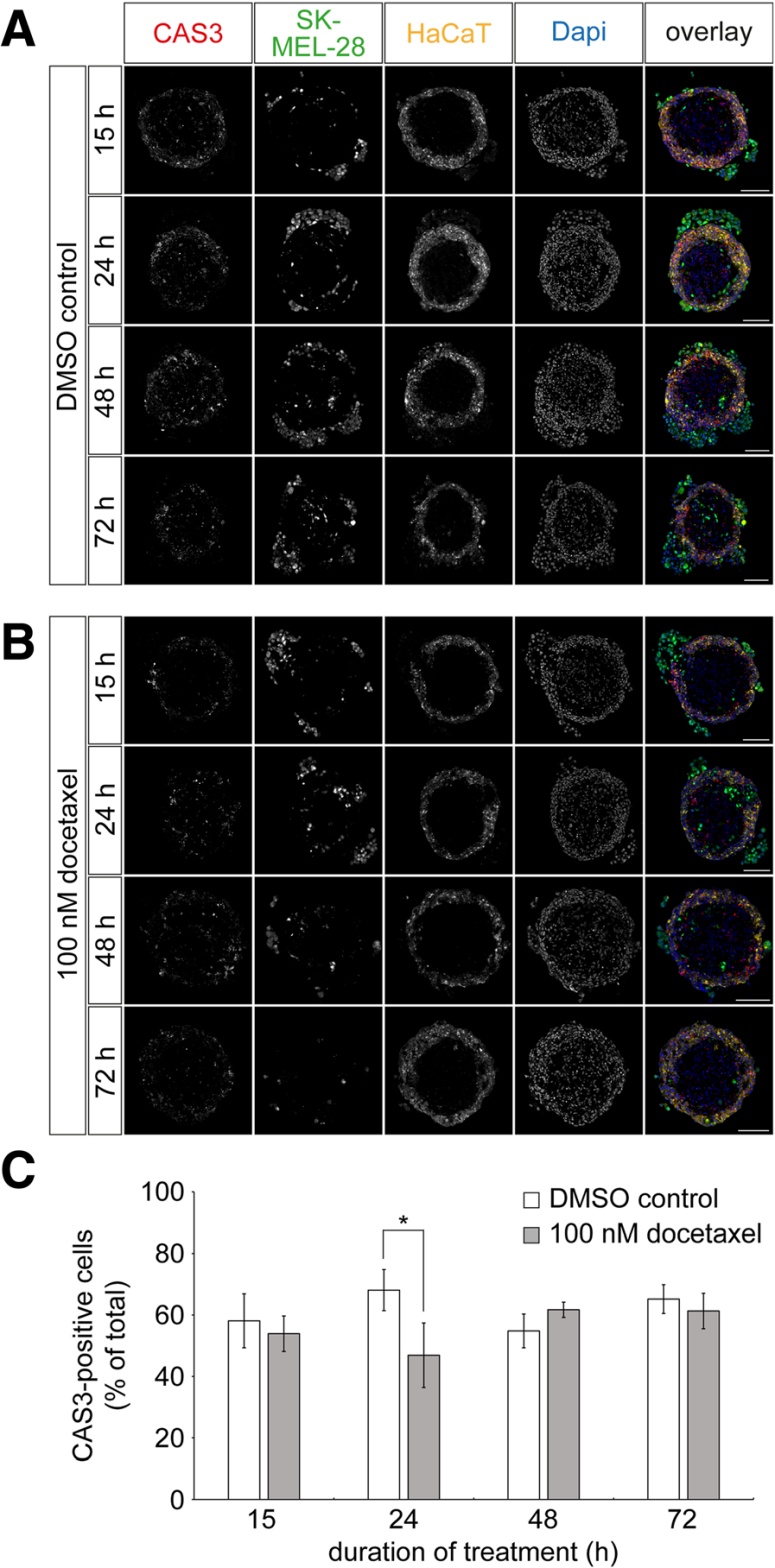
Loss of external melanoma cells upon docetaxel treatment hampers mechanistic explanation of drug effects. Tri-culture spheroids were generated by 3D cultivation of fibroblasts for three days, followed by simultaneous addition of keratinocytes and melanoma cells. HaCaT and SK-MEL-28 cells were labeled with CellTrackerRed CMPTX and CellTrackerGreen CMFDA dyes, respectively. After another two days, tri-culture spheroids were treated with 0.01 ‰ of DMSO as control or 100 nM docetaxel for 15, 24, 48, and 72 h. Spheroids were cryosectioned into 10-μm thick slices and stained for CAS3. **a** and **b** Representative confocal images of control (**a**) and docetaxel-treated cultures (**b**). In overlay images, CAS3 immunostaining signals, SK-MEL-28, HaCaT, and nuclei are depicted in red, green, yellow, and blue, respectively. Scale bars: 100 μm. (**C**) Quantification of CAS3-positive cells. Graph displays the amounts of CAS3-positive cells as mean ± SEM (n ≥ 3 independent experiments; * *P* < 0.05) in percent of the whole cell count per slice. For each experiment and time point, ≥ 3 spheroids were analyzed

We next investigated the effect of docetaxel on the apoptosis of tri-culture spheroids over time. Therefore, tri-cultures were incubated with solvent control or docetaxel as before and harvested after 15, 24, 48, and 72 h of treatment. Cryosections were made and stained for CAS3. Figure 4 a-b shows representative confocal microscopy images of spheroids treated with solvent control (Fig. 4a) or docetaxel (Fig. 4b). The general morphology of tri-cultures with increasing and decreasing numbers of external melanoma cells in the control and docetaxel-treated samples, respectively, was as observed before. Yet, quantitative analysis showed that the fraction of CAS3-positive cells in the entire tri-culture was apparently unaltered by docetaxel. Only at 24 h of treatment, a significant difference was found with 68.1% ± 6.7% (mean ± SEM, *n* = 3 independent experiments, Fig. 4c) compared to 46.9% ± 10.5% (mean ± SEM, *n* = 4 independent experiments, Fig. 4c) of CAS3-positive cells in the absence versus presence of docetaxel. This result suggested that apoptosis was either not involved in the drug response or that technical constraints of the model might have blurred this information.

### A modified preparation avoids docetaxel-induced loss of external SK-MEL-28 cells

**Fig. 5:**
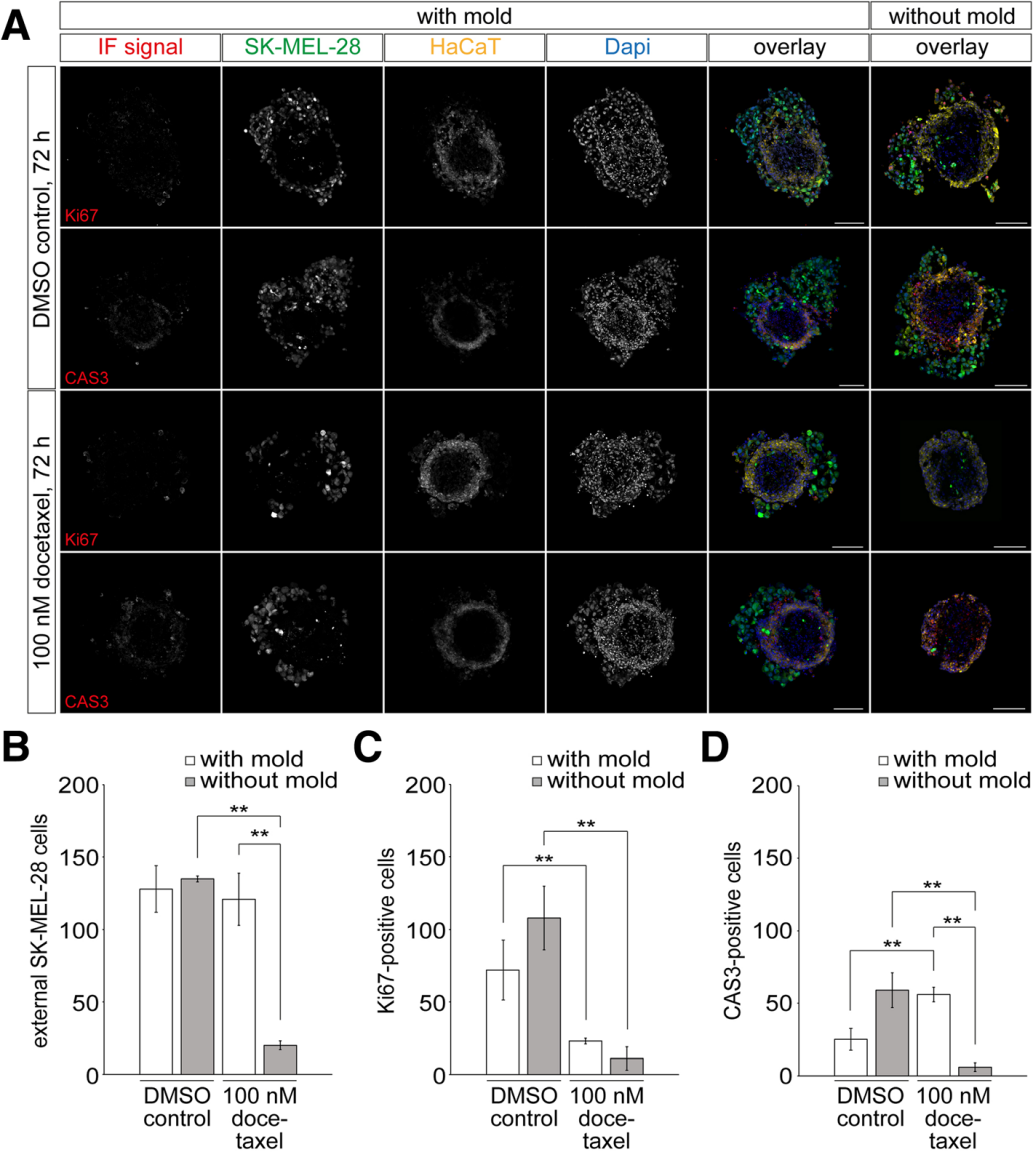
Processing of tri-cultures in special agarose molds reveals docetaxel-induced increase of apoptosis and reduction of proliferation of external melanoma cells. Tri-culture spheroids were generated by 3D cultivation of fibroblasts for 3 days, followed by simultaneous addition of keratinocytes and melanoma cells. HaCaT cells were labeled with CellTrackerRed CMPTX dye and SK-MEL-28 cells with CellTrackerGreen CMFDA dye. For the mold technique, spheroids were transferred on day five into 3D-agarose molds and then treated with 0.01 ‰ of DMSO as control or 100 nM docetaxel in DMSO for 72 h. Washing and embedding for cryosectioning occurred in the molds, as well. For the samples without mold, spheroids handled as in Figs. 3 and 4, i.e. they were treated in the cell repellent plate and then transferred to an Eppendorf tube for washing and embedding. Subsequently, all spheroids were cryosectioned into 20-μm thick slices and stained for either Ki67 or CAS3 as indicated (**a**). Scale bars: 100 μm. **b-d** Quantification of total numbers of external SK-MEL-28 cells (**b**), as well as amounts of Ki67- (**c**) and CAS3-positive external SK-MEL-28 cells (**d**) of tri-culture spheroids processed with or without mold. Graph displays mean ± SEM (n ≥ 3 independent experiments; ** P < 0.01). For each experiment, ≥ 3 spheroids were analyzed

Apparently, treatment of tri-culture spheroids with docetaxel led to an ablation of external SK-MEL-28 cells. To understand the fate of those cells, we performed experiments using a 3D-mold technique. Reasoning that docetaxel might weaken cell-cell interactions and that many of the affected cells might have been lost in the previous assays where tri-cultures were transferred by pipetting from the culture to a staining/washing station after docetaxel treatment (for a scheme, see Additional file 1: Figure S1B), we here tried to avoid any post-treatment stress to the samples. Thus, tri-culture spheroids were first grown as before in cell-repellent plates, but were then transferred on day five into an agarose 3D mold (Additional file 1: Figure S1C). Treatment with docetaxel and all following processing steps were carried out in these molds. Indeed, the entire molds with the treated spheroids inside were embedded in OCT, cryosectioned, and slices were then stained for nuclei and Ki67 or CAS3. Figure 5a shows a comparison of representative confocal images of the tri-culture spheroids processed with both techniques, i.e. agarose mold (‘with mold’, left panels) and standard transfer washing/staining station technique (‘without mold’, right panel). As shown in Fig. 5a, the majority of external melanoma cells were lost or still present upon docetaxel treatment when processed without or within molds, respectively. This was confirmed by quantitative analysis of external melanoma cells, which revealed significant differences in the numbers of external melanoma cells between the two techniques (Fig. 5b). Upon processing without the molds, docetaxel treatment resulted in a significant decrease of the number of external SK-MEL-28 cells from 135 ± 2 to 20 ± 3 (mean ± SEM, *n* ≥ 3 independent experiments, Fig. 5b). Conversely, when processed within the molds, the number of external melanoma cells remained unchanged after docetaxel treatment (Fig. 5b). With respect to the number of proliferating cells, the processing technique had less impact. Indeed, in both, with and without the mold, docetaxel treatment led to a significant drop of the Ki67-positive numbers of external melanoma cells (Fig. 5c). However, the processing was relevant when addressing the fraction of apoptotic cells. Using the processing without mold, the number of CAS3-positive external melanoma cells decreased from 59 ± 12 cells (mean ± SEM, *n* = 3 independent experiments) to 6 ± 3 cells (mean ± SEM, *n* = 4 independent experiments) in the absence versus presence of docetaxel (Fig. 5d). Conversely, when processed within the molds, the number of CAS3-positive external SK-MEL-28 cells significantly increased from 25 ± 6 (mean ± SEM, n = 4 independent experiments) with DMSO to 56 ± 5 (mean ± SEM, n = 4 independent experiments) with docetaxel (Fig. 5d). In summary, these data suggest that proper post-treatment processing is essential for the interpretation of the behavior of external melanoma cells and that a combination of reduced proliferation and increased apoptosis is induced by docetaxel treatment. Furthermore, docetaxel-induced apoptosis can, at least partially, explain the loss of external melanoma cells in the tri-culture model.

### Docetaxel treatment of tri-culture spheroids restores keratinocyte differentiation

**Fig. 6:**
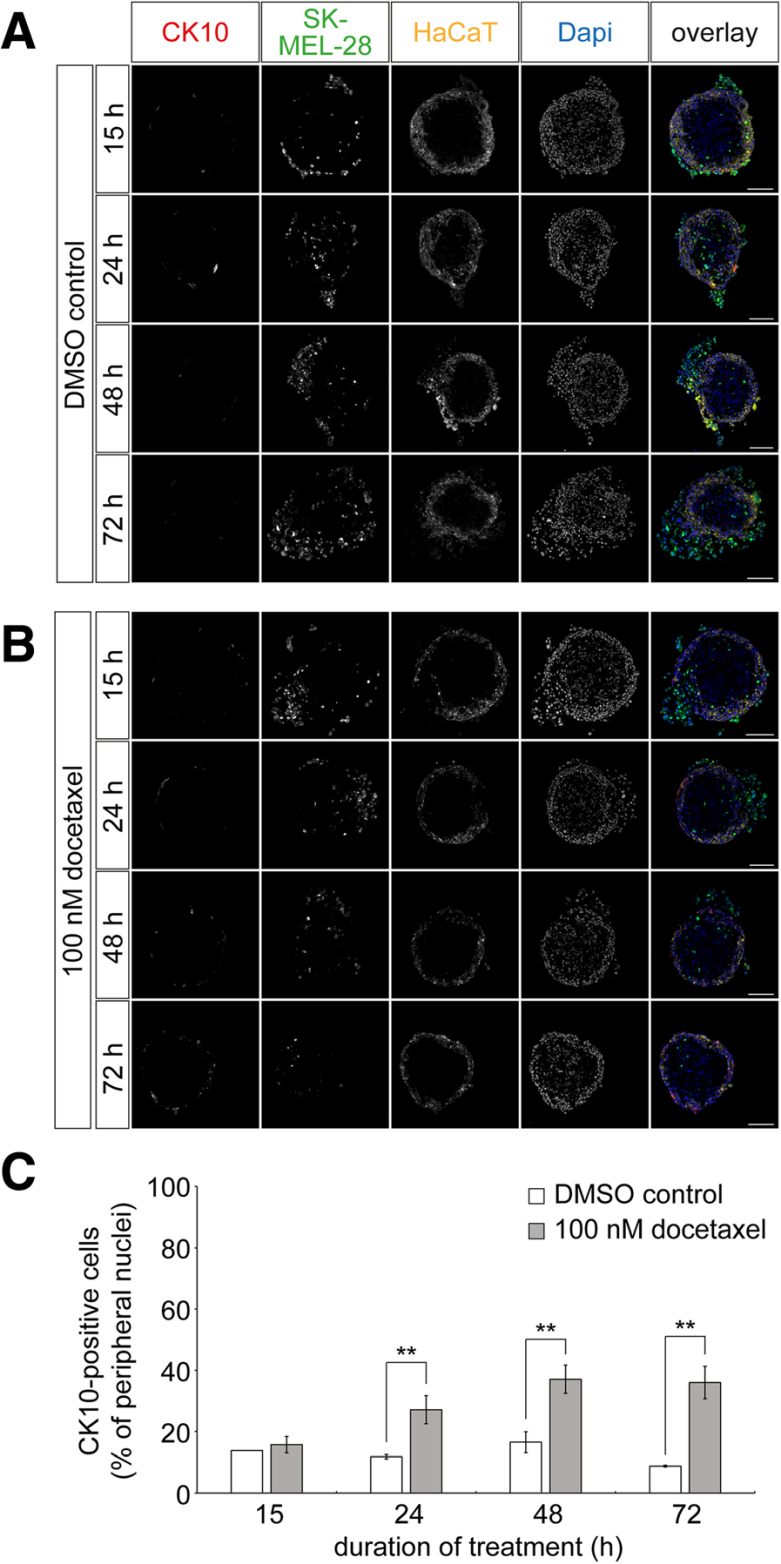
Melanoma cells impair expression of keratinocyte differentiation marker CK10. Tri-culture spheroids were generated by 3D cultivation of fibroblasts for three days, followed by simultaneous addition of keratinocytes and melanoma cells. HaCaT and SK-MEL-28 cells were labeled with CellTrackerRed CMPTX dye and CellTrackerGreen CMFDA, respectively. After another two days, tri-culture spheroids were treated with 0.01 ‰ of DMSO as control or 100 nM docetaxel for 15, 24, 48, and 72 h. Spheroids were cryosectioned into 10-μm thick slices and stained for CK10. (**A and B**) Representative confocal images of control (**A**) and docetaxel-treated cultures (**B**). In overlay images, CK10 immunostaining signals, SK-MEL-28, HaCaT, and nuclei are depicted in red, green, yellow, and blue, respectively. Scale bars: 100 μm. (**C**) Quantification of CK10-positive cells. Graph displays the amounts of CK10-positive cells as mean ± SEM (n ≥ 3 independent experiments; ** P < 0.01) in percent of the peripheral nuclei per slice. For each experiment and time point, ≥ 3 spheroids were analyzed

Previously, it was observed in human malignant melanoma biopsies that neoplastic cells hamper keratinocyte differentiation [13]. To address this finding in our 3D-melanoma model, we stained cryosections of tri-culture spheroids with an antibody against CK10 in the absence and presence of docetaxel. Figure 6a-b shows representative confocal images of these samples. In control-treated spheroids, an increase in the number of external SK-MEL-28 cells as well as a low level of the keratinocyte differentiation marker CK10 were observed throughout the experiment time course (Fig. 6a). In contrast, treatment with docetaxel resulted in a restoration of CK10 expression that occurred concomitant to the ablation/apoptosis of external melanoma cells (Fig. 6b). Quantitative analysis revealed that the number of peripheral CK10-positive cells was significantly higher in docetaxel treated spheroids as compared to controls, beginning at 24 h of treatment until the end of the observation period (Fig. 6c). Treated tri-culture spheroids reached a number of CK10-positive cells of 36% ± 5% (mean ± SEM, *n* = 4 independent experiments, Fig. 6c). In summary, this suggests that the tri-culture model is able to reflect effects of melanoma cells on keratinocyte differentiation as observed in human disease and that such loss of differentiation can be restored by treatment with docetaxel.

### Docetaxel treatment leads to augmented ABCB5-signals in external melanoma cells

**Fig. 7:**
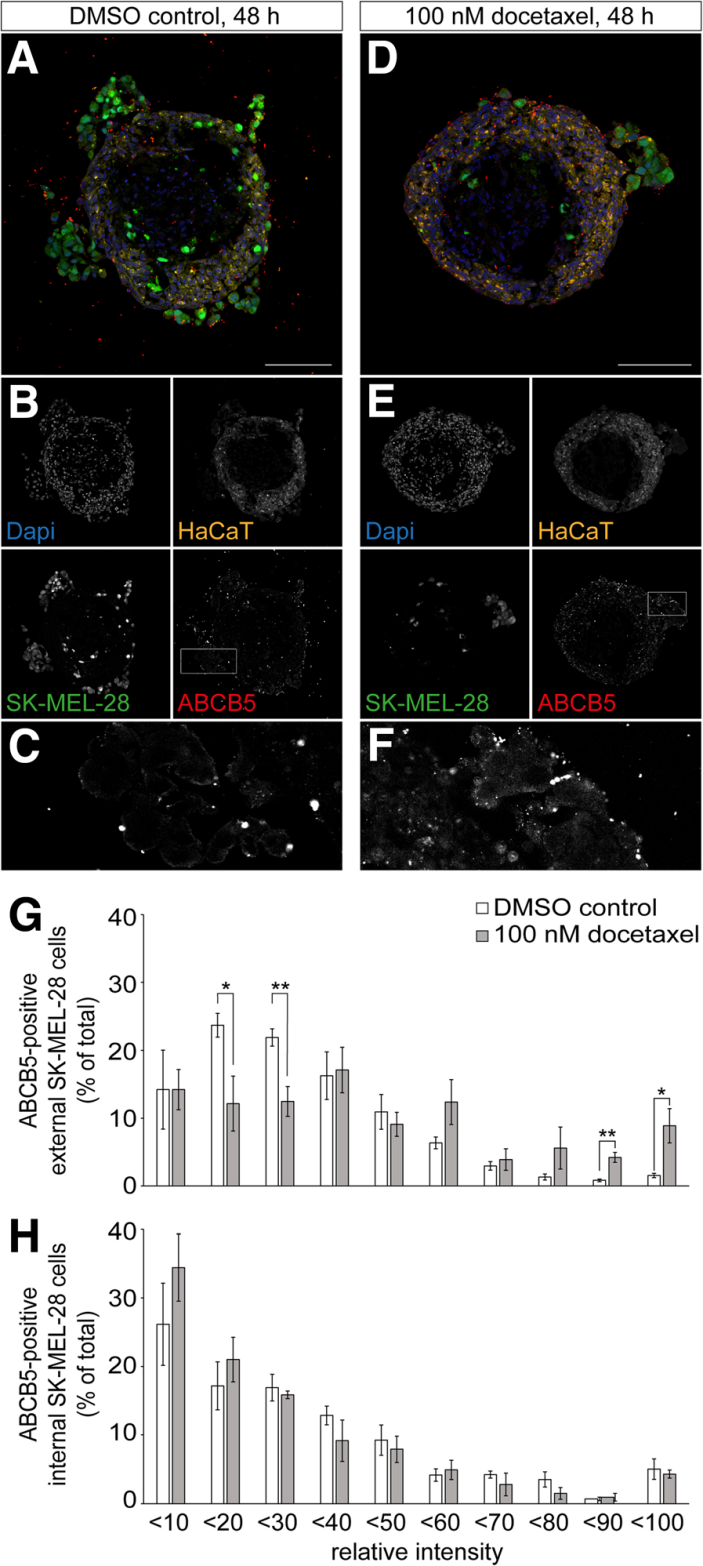
Cytostatic treatment leads to enhanced ABCB5-signals in keratinocytes and external melanoma cells. Tri-culture spheroids were generated by 3D cultivation of CCD-1137Sk cells for three days, followed by the combined addition of HaCaT and SK-MEL-28 cells. HaCaT cells were labeled with CellTrackerRed CMPTX dye and SK-MEL-28 cells with CellTrackerGreen CMFDA dye. After another two days, tri-culture spheroids were treated with 0.01 ‰ of DMSO as control (**a-c**) or 100 nM docetaxel in DMSO (**d-f**) for 48 h. Spheroids were cryosectioned into 10-μm thick slices and immunostained for mouse anti-ABCB5 from TICEBA. **a** and **d** Overlay images of the confocal sections shown in **b** and **e**. In overlays, ABCB5 signals, melanoma cells, keratinocytes, and nuclei are depicted in red, green, yellow, and blue, respectively. Scale bars: 100 μm. **c** and **f** Detail images of ABCB5 signals from boxed regions in **b** and **e**. **g-h** Quantification of the relative intensity of ABCB5-positive external (**g**) and internal (**h**) SK-MEL-28 cells (percentage of total). Given is mean ± SEM (*n* = 4 independent experiments; * P < 0.05, ** P < 0.01). For each experiment, ≥ 3 spheroids were analyzed

Considering the multidrug resistance capabilities of ABCB5 for doxorubicin and temozolomide in melanoma cells [23, 24], we wondered whether there would be a correlation between ABCB5 expression and melanoma cell survival to drug treatment also in the tri-culture model. Therefore, tri-cultures were exposed to 100 nM of docetaxel or DMSO for 48 h, fixed, sliced, and then stained for ABCB5 with m3C2-1D12 [23] primary antibody. Surface expression of ABCB5 and specificity of the antibody on SK-MEL-28 cells were proven using flow cytometry and competitive peptide analyses in immunofluorescence (Additional file 3: Figure S3). As depicted in Fig. 7a-f, mostly melanoma cells as well as keratinocytes showed ABCB5 immunoreactivity. Quantitative analysis confirmed an increase in the number of external melanoma cells with high ABCB5 immunofluorescence intensity upon drug treatment (Fig. 7g), while internal melanoma cells were apparently unaffected in that respect (Fig. 7h). Interestingly, also keratinocytes displayed an increase in ABCB5 immunofluorescence upon docetaxel treatment (compare Fig. 7b and e). The findings regarding the drug-induced enrichment of strongly ABCB5-positive external SK-MEL-28 and HaCaT cells as well as the lack of effect on internal melanoma cells were confirmed by another anti-ABCB5 antibody (Additional file 4: Figure S4). In summary, these data are consistent either with docetaxel-induced enhancement of ABCB5 expression in weakly expressing cells or selection of cells with high ABCB5 levels.

## Discussion

3D in vitro models of melanoma are increasingly used to study drug efficacy and mode of action as well as drug combinations. Compared to classical two-dimensional cell cultures, 3D models are thought to better represent a series of parameters that are critical for cancer cell behavior, including substrate stiffness, cell-cell interactions, distribution of oxygen and waste products, as well as drug diffusion [36]. Currently existing 3D models are mostly tuned for either simplicity and high-throughput, complexity and similarity to the in vivo situation, or personalized medicine [31, 32, 36–43]. Here, we aimed to set up an early-stage 3D melanoma model that would allow to investigate several relevant drug-induced processes in a quantitative and cell-type specific manner. Yet, it should be also fast, easy, and robust in performance. The solution presented in this study is an easy to handle spheroid-based model composed of melanoma cells and major cellular components of a stroma-like environment, i.e. human fibroblasts and keratinocytes. To avoid batch-to-batch variability and to render the system cost effective, we opted for the established cell lines SK-MEL-28, HaCaT, and CCD-1137Sk. Within these constraints, the model was found to reliably mimic melanoma cell invasion into the dermal compartment, drug-induced selection of ABCB5-expressing melanoma cells, and loss of melanoma-induced keratinocyte differentiation.

As for the latter, optimal differentiation of keratinocytes in vitro leading to human skin equivalents (HSE), typically requires the use of primary cells and multifactorial external control, including pH and Ca^2+^ gradients as well as air lift [38, 41, 42]. Given that these operations are time consuming and difficult to generate in high numbers, we avoided such complex maneuvers and allowed HaCaT cells to automatically stratify on top of a fibroblast core. Compared to HSE models, we observed a partial differentiation pattern that included stratification into lower and upper strata expressing either CK14 or CK10, respectively, but lacked a cornified layer. However, while HSE models often come with generation times of several weeks [44], the spheroid-based tri-culture was ready for use after only seven days. It is arguable, whether the observed stratification really reflects differentiation or if pre-differentiated cells migrated to the outer regions of the spheroid. Yet, we observed an interesting related feature that is also known from human melanoma. Indeed, melanoma cells were reported to influence the differentiation pattern of human epidermal keratinocytes in vivo, i.e. that it leads to a loss of CK10 in hyperplastic regions [13]. Consistent with this, we found that melanoma cells also decreased CK10 expression by HaCaT cells in our tri-culture spheroids (Figs. 2 and 6). Notably, such loss of CK10 expression mostly occurred in direct vicinity of melanoma cells and keratinocyte differentiation was restored upon treatment with docetaxel which led to apoptosis of external melanoma cells (Fig. 6).

Another interesting feature of the present model was the division of melanoma cells into two populations, i.e. external and internal. The finding of internal SK-MEL-28 cells suggested their invasion into the fibroblast core and this would fit to the fact that this cell line is from the metastatic phase of melanoma [37] and known to rapidly migrate downwards through the skin [45]. In general, it would be interesting to further explore these cultures as a simple test system for antimigratory effects of diverse drugs. Apart from these future prospects, the identification of two melanoma cell pools was also interesting, because both pools showed differential behavior in at least three characteristics. First, external melanoma cells, which were located on the outside of the spheroids and thus in direct contact to keratinocytes, tended to form growing aggregates. Conversely, internal melanoma cells, which were found in the fibroblast core, were typically solitary and did not coalesce (Fig. 2). Apart from the first day after adding the keratinocyte-melanoma cell mixture to the fibroblast core, melanoma cells were hardly ever found in the HaCaT ring but always in the fibroblast core (Additional file 5: Figure S5). The second clear difference between internal and external melanoma cells was their response to docetaxel. While external cells massively went into apoptosis and became loose, internal melanoma cells remained apparently unaffected. Their numbers were stable even after 72 h of treatment and the relative amounts of apoptotic and proliferating cells was unaltered. It would be interesting to know whether such differential behavior was due to limited access of the drug to the spheroid core or rather due to cell-specific differences. For example, it could be that only drug-resistant cell subpopulations, which are frequently observed in malignant melanoma [46], were able to invade the fibroblast core or whether some cellular signaling within the core would have led to drug insensitivity. In any case, it was intriguing to observe that docetaxel changed proliferation and apoptosis apparently only in melanoma cells but not in keratinocytes or fibroblasts. A third difference between external and internal melanoma cells was related to their expression of the ATP-dependent transporter protein, ABCB5. Based on ABCB5 immunofluorescence signal intensity profiles, docetaxel led to higher ABCB5 immunofluorescence signals in the external but not the internal melanoma cells (Fig. 7 and Additional file 4: Figure S4). We do not think that internal melanoma cells would have been unable to increase ABCB5 expression, because – contrary to our expectation – they typically showed lower ABCB5 signals than external ones before treatment. Thus, it could again be that internal melanoma cells either represented a special subpopulation of cells, or they were not exposed to sufficient amounts of the drug, or their local microenvironment impaired such drug-induced changes in gene expression. However, the observed effect of docetaxel on ABCB5 signals in external melanoma cells is compatible with either, an up-regulation of ABCB5 in weakly expressing cells or selection of strongly expressing cells. In general, our results fit nicely to previous studies, which reported that ABCB5 expression is enhanced in malignant melanoma [47], that it has a functional role in tumor growth [48], and that chemotherapy leads to the selection of ABCB5-expressing cells [24].

For the present study, docetaxel was used as a test substance. Although mitogen-activated protein kinase pathway inhibitors and immunotherapies against the immune checkpoints cytotoxic T lymphocyte-associated antigen and programmed death 1 have largely replaced classical alkylating and cytostatic chemotherapeutics as first-line treatment [20, 22], the mitotic inhibitor paclitaxel and its derivative docetaxel [49] are being considered as adjuvant treatments [19, 21, 22] and explored for use in novel formulations (see e.g. [34, 35, 50, 51]). Given that our model in its current version lacks immune cells, immunotherapies were not in the focus of this study. While the addition of T-cells and other immune cell components to future adaptations of the present 3D tri-culture might be valuable ideas to follow, we here concentrated on the effects of a classical agent on melanoma cells and on their chemoresistance features. With respect to effective drug concentrations, significant effects on external SK-MEL-28 survival were observed after 48 h at 100 nM of docetaxel. In comparison, SK-MEL-28 cells cultured in 2D appeared much more susceptible to docetaxel treatment (Additional file 2: Figure S2). This is in agreement with previous studies, which found a maximal effect of docetaxel at around 10–20 nM on different 2D melanoma cell cultures [52] and a generally altered sensitivity of cells grown in 2D versus 3D [53–55].

In comparison with the present study, other three-dimensional melanoma spheroid models using, for example, the liquid overlay method [56] are only composed of one cell type, the melanoma cells. Thus, they do not aim to represent the stromal environment of a tumor. On the other hand, HSE models are often generated by seeding primary fibroblasts in collagen type I followed by simultaneous seeding of primary keratinocytes together with melanoma cells [57] or by seeding melanoma cells with primary fibroblasts to embed both cell types in the collagen type I matrix [44]. This method spontaneously forms melanoma nests. Therefore, numbers and sizes of such nests might vary between individual skin reconstructs. As a consequence, it is often difficult to quantitatively validate these models and to predict therapeutic impacts. Conversely, the present tri-culture spheroid model always formed very similar sizes of spheroids with a highly reproducible arrangement of the different cell types allowing reliable quantification of cellular drug effects. Next, skin-on-a-chip models can be performed under a controlled perfusion of growth factors or nutrients [58]. This cannot be realized in a static spheroid-based system as presented here. Using a skin-on-a-chip platform, Abaci and co-workers demonstrated that the cancer drug, doxorubicin, may have direct toxic effects on keratinocyte proliferation and differentiation [59]. However, this platform is not suitable for high-throughput screening. For this purpose, simple spheroid models might be more appropriate. Given that the tri-culture spheroid model is composed of both, stroma and tumor cells, it is also possible to test general cell toxicity of a drug by evaluating the effect on surrounding non-transformed cells [60].

On a technical note, the docetaxel treatment of tricultures led to a consistent loss of external melanoma cells. However, this was only true when the cultures were transferred after treatment from the spheroid formation plate into another container for washing (Fig. 5). If docetaxel treatment, washing, and embedding were carried out altogether without any transfer, presumably all – dead and alive – cells were still present in the immediate vicinity of the spheroids. Although it cannot be completely excluded that the observed difference in cell numbers was due to a distinct effect of the drug in the agarose mold versus the plastic plate, the most straight forward explanation appears to be that many of the external melanoma cells became loose upon drug treatment and were lost during the transfer from one container to the next due to mechanical shear force (see Additional file 1: Figure S1B for schematic illustration). This finding might be of general interest, because similar mechanisms of treatment-induced cell loss could possibly also occur in other spheroid or organoid models. If phenotypic quantitative analysis of either culture size, cell number, or fraction of apoptotic or proliferating cells is used, this effect could easily lead to erroneous data interpretation. Clearly, further investigation in that direction would be useful.

## Conclusions

In the present study, a convenient spheroid-based tri-culture melanoma model was established. This model is composed of fibroblasts, keratinocytes, and melanoma cells that arrange in a highly reproducible and quantifiable manner in 3D. Melanoma cell invasion into the fibroblast core, melanoma-induced loss of keratinocyte differentiation, and cell-type specific drug responses to docetaxel were described as major hallmarks of the model. Future applications might consider addition of further cell types, including immune or primary cells, to further expand the applicability of such a system for the screening of drug candidates and their modes of action.

## Additional files

Additional file 1: **Figure S1.** Transfer of docetaxel-treated tri-cultures leads to massive loss of external melanoma cells. Drawings schematically depicting the general composition of the tri-culture model (**A**) and the proposed mechanism for the loss of external SK-MEL-28 cells upon docetaxel treatment (**B**). (**A**) A core of CCD-Sk1137 fibroblasts (grey) is surrounded by a ring of CK14-positive HaCaT keratinocytes (yellow), and this by CK10-positive HaCaT keratinocytes (red). SK-MEL-28 melanoma cells (green) can be divided in individual ‘internal’ melanoma cells found largely in the fibroblast core, and clustered ‘external’ melanoma cells located on the outer rim of the tri-cultures. (**B**) In all experiments, spheroid formation was performed in cell repellent plates. In mold experiments (left part), spheroids were then transferred to an agarose mold, where docetaxel treatment was followed by washing and embedding for cryosectioning. Subsequently, cryosections were immunostained. In experiments without mold, docetaxel treatment was also done in the cell repellent plate. Then, treated spheroids were transferred to another standard plastic well for washing and embedding. Presumably, external melanoma cells got loose upon docetaxel treatment and were largely lost upon transfer in the experiments without mold. This is schematically shown by the loosened cells in the pipette on the right side of the scheme. (**C**) Micrograph of a tri-culture spheroid in the agarose mold. Note, that the agarose does not cover the spheroid, thus, docetaxel can freely access the spheroid as in the standard plastic well. The advantage of the mold is, that it can be directly cryosectioned avoiding further steps of pipetting. (JPG 1420 kb)

Additional file 2: **Figure S2.** Comparison of SK-MEL-28 response to docetaxel in 2D versus 3D**.** 2D cultures of SK-MEL-28 cells were grown up to 50% of confluency. Tri-culture spheroids were produced by 3D cultivation of fibroblasts for 3 days, followed by the combined addition of keratinocytes and melanoma cells, and another 2 days without treatment. Then, all cultures were treated with different concentrations of docetaxel for 24 h (2D) or 48 h (spheroids). Spheroids were cryosectioned into 10-μm-thick slices, 2D cultures were directly fixed. Subsequently, all samples were labeled with Dapi and then imaged by confocal microscopy. The numbers of remaining SK-MEL-28 cells (2D cultures) or of external SK-MEL-28 cells (spheroids) were determined. The graph shows the amounts of SK-MEL-28 cells as a function of docetaxel concentration and normalized to the control condition without docetaxel. Given is mean ± SEM (*n* ≥ 3; * *P* < 0.05, ** *P* < 0.01). (JPG 173 kb)

Additional file 3: **Figure S3.** Specificity of m3C2 anti-ABCB5 antibody on SK-MEL-28 cells is proven by FACS and immunofluorescence methods. (**A-D**) SK-MEL-28 cells were analyzed for surface expression of ABCB5 by incubation of 2.5 × 10^5^ cells for 30 min at 4 °C with m3C2-1D12 anti-ABCB5 antibody or MOPC-31C mouse isotype control antibody (10 μg/ml). This was followed by incubation with FITC-conjugated goat anti-mouse secondary antibody (PharMingen) and single-color flow cytometry. Panels depict cytometry-scatter plots of unstained (**A**), only secondary-antibody stained (**B**), isotype plus secondary-antibody stained (**C**), or anti-ABCB5 plus secondary-antibody stained samples (**D**). Gate C was used to count ABCB5-positive cells. This contained 0.34% ± 0.15% (mean ± SD) and 6.64% ± 1.46% (mean ± SD) of cells in C and D, respectively. (**E-H**) Specificity of m3C2-1D12 anti-ABCB5 antibody on immunofluorescence of SK-MEL-28 cells was tested using standard protocols in the presence of FITC-conjugated secondary antibody only (**E**) or of m3C2-1D12 plus FITC-conjugated secondary antibody (**F-H**). In addition, primary antibody binding was competed by incubation of 2 μM ABCB5 epitope peptide (**F**) or scrambled peptide (**G**). Scale bar: 20 μm. (JPG 962 kb)

Additional file 4: **Figure S4.** Enhancement of ABCB5-signals in keratinocytes and external melanoma cells upon docetaxel treatment is confirmed by a second anti-ABCB5 antibody. Tri-culture spheroids were generated by 3D cultivation of CCD-1137Sk cells for 3 days, followed by the combined addition of HaCaT and SK-MEL-28 cells. HaCaT and SK-MEL-28 cells were labeled with CellTrackerRed CMPTX and CellTrackerGreen CMFDA dye, respectively. After another 2 days, tri-culture spheroids were treated with 0.01 ‰ of DMSO as control (**A-C**) or 100 nM docetaxel in DMSO (**D-F**) for 48 h. Spheroids were cryosectioned into 10-μm thick slices and immunostained with mouse anti-ABCB5 antibody MA5–17026. (**A and D**) Overlay images of the confocal sections shown in B and E. In overlays, ABCB5 signals, melanoma cells, keratinocytes, and nuclei are depicted in red, green, yellow, and blue, respectively. Scale bars: 100 μm. (C and F) Detail images of ABCB5 signals from boxed regions in B and E. (G-H) Quantification of the relative intensity of ABCB5-positive external (G) and internal (H) SK-MEL-28 cells (percentage of total). Given is mean ± SEM (*n* = 4 independent experiments; * *P* < 0.05, ** *P* < 0.01). For each experiment, ≥ 3 spheroids were analyzed. (JPG 1327 kb)

Additional file 5: **Figure S5.** Accumulation of external melanoma cells in tri-cultures is an active separation process. Tri-culture spheroids were generated by 3D cultivation of fibroblasts for 3 days, followed by simultaneous addition of keratinocytes and melanoma cells. HaCaT and SK-MEL-28 cells were pre-labeled with CellTrackerRed CMPTX and CellTrackerGreen CMFDA dyes, respectively. After another one (‘day 4’, upper row) or 2 days (‘day 5’, lower panels), tri-culture spheroids were cryosectioned into 10-μm thick slices and stained with Dapi. Representative confocal images are shown. While most melanoma cells were embedded in the keratinocyte ring on day four, they segregated from keratinocytes on day five and either accumulated in the periphery of the culture (‘external’ melanoma cells) or within the fibroblast core (‘internal’ melanoma cells). The fibroblast core is located in the center of the tri-culture and identified as Dapi-positive plus CellTracker-negative. Scale bars: 100 μm. (JPG 467 kb)

## Abbreviations

ABCB5: ATP-binding cassette transporter type B5
ANOVA: analysis of variance
BRAF: gene encoding proto-oncogene B-Raf
CAS3: cleaved caspase 3
CK10: cytokeratin 10
CK14: cytokeratin 14
DMSO: dimethylsulfoxide
HSE: human skin equivalent
Ki67: Antigen Ki-67
