# Supplementary figures and images for: A novel spheroid-based co-culture model mimics loss of keratinocyte differentiation, melanoma cell invasion, and drug-induced selection of ABCB5-expressing cells
Source: BMC Cancer. 2019 Apr 29;19:402. doi: 10.1186/s12885-019-5606-4 (PMC6489189; doi:10.1186/s12885-019-5606-4)

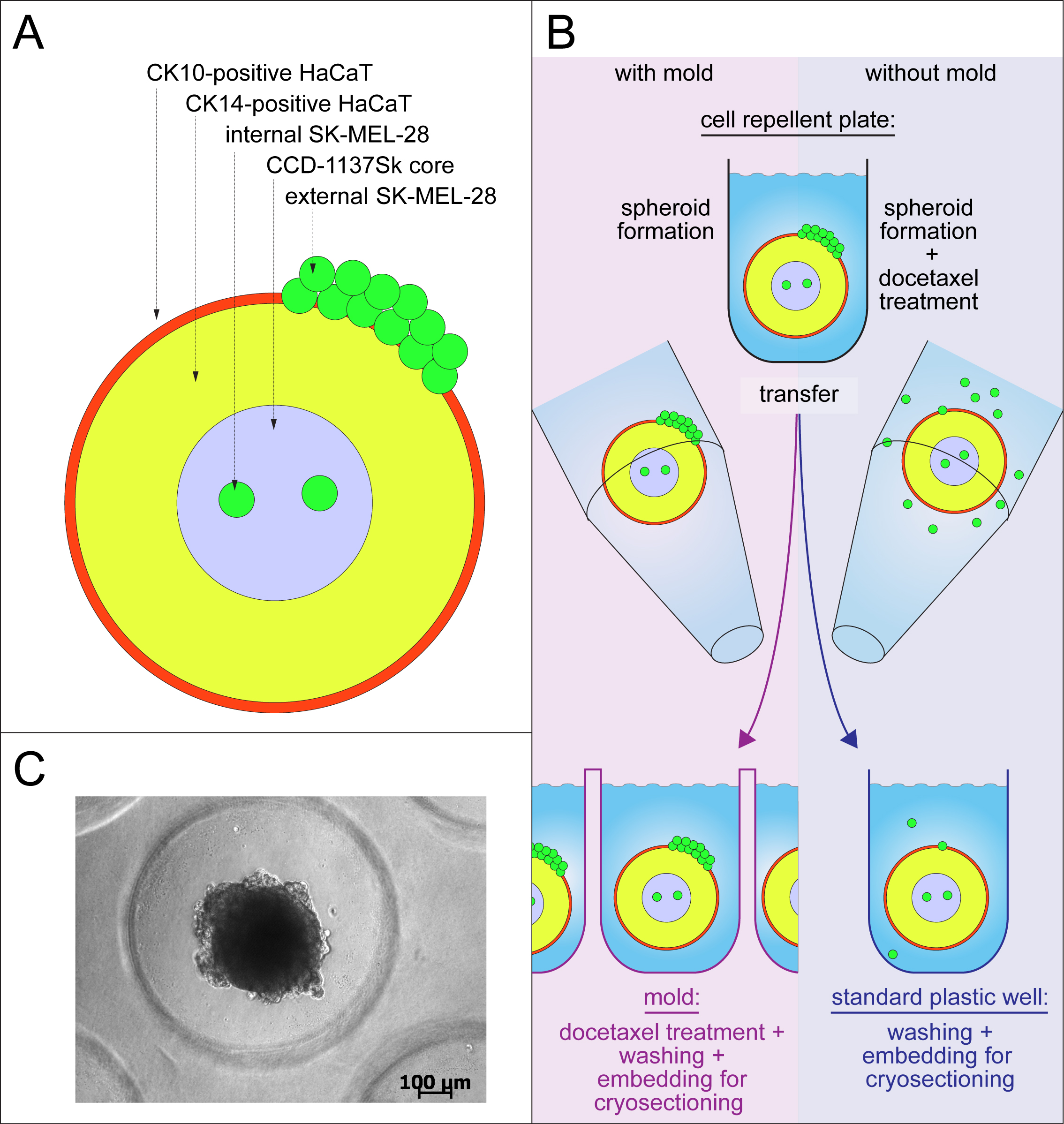

Supplement: Supplementary file 1 — Figure S1. Transfer of docetaxel-treated tri-cultures leads to massive loss of external melanoma cells. Drawings schematically depicting the general composition of the tri-culture model (A) and the proposed mechanism for the loss of external SK-MEL-28 cells upon docetaxel treatment (B). (A) A core of CCD-Sk1137 fibroblasts (grey) is surrounded by a ring of CK14-positive HaCaT keratinocytes (yellow), and this by CK10-positive HaCaT keratinocytes (red). SK-MEL-28 melanoma cells (green) can be divided in individual ‘internal’ melanoma cells found largely in the fibroblast core, and clustered ‘external’ melanoma cells located on the outer rim of the tri-cultures. (B) In all experiments, spheroid formation was performed in cell repellent plates. In mold experiments (left part), spheroids were then transferred to an agarose mold, where docetaxel treatment was followed by washing and embedding for cryosectioning. Subsequently, cryosections were immunostained. In experiments without mold, docetaxel treatment was also done in the cell repellent plate. Then, treated spheroids were transferred to another standard plastic well for washing and embedding. Presumably, external melanoma cells got loose upon docetaxel treatment and were largely lost upon transfer in the experiments without mold. This is schematically shown by the loosened cells in the pipette on the right side of the scheme. (C) Micrograph of a tri-culture spheroid in the agarose mold. Note, that the agarose does not cover the spheroid, thus, docetaxel can freely access the spheroid as in the standard plastic well. The advantage of the mold is, that it can be directly cryosectioned avoiding further steps of pipetting. (JPG 1420 kb) [file 12885_2019_5606_MOESM1_ESM.jpg]

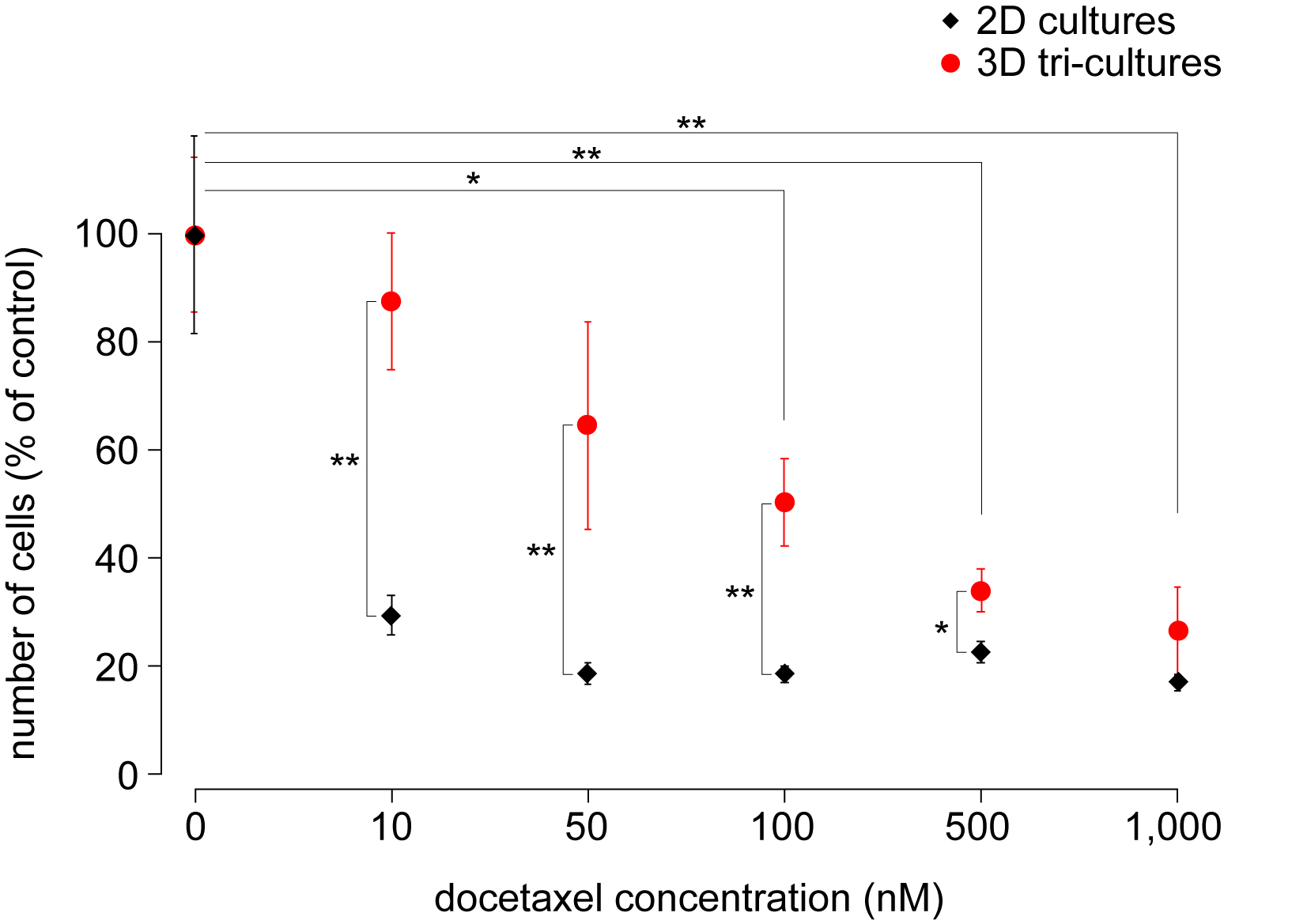

Supplement: Supplementary file 2 — Figure S2. Comparison of SK-MEL-28 response to docetaxel in 2D versus 3D. 2D cultures of SK-MEL-28 cells were grown up to 50% of confluency. Tri-culture spheroids were produced by 3D cultivation of fibroblasts for 3 days, followed by the combined addition of keratinocytes and melanoma cells, and another 2 days without treatment. Then, all cultures were treated with different concentrations of docetaxel for 24 h (2D) or 48 h (spheroids). Spheroids were cryosectioned into 10-μm-thick slices, 2D cultures were directly fixed. Subsequently, all samples were labeled with Dapi and then imaged by confocal microscopy. The numbers of remaining SK-MEL-28 cells (2D cultures) or of external SK-MEL-28 cells (spheroids) were determined. The graph shows the amounts of SK-MEL-28 cells as a function of docetaxel concentration and normalized to the control condition without docetaxel. Given is mean ± SEM (n ≥ 3; * P < 0.05, ** P < 0.01). (JPG 173 kb) [file 12885_2019_5606_MOESM2_ESM.jpg]

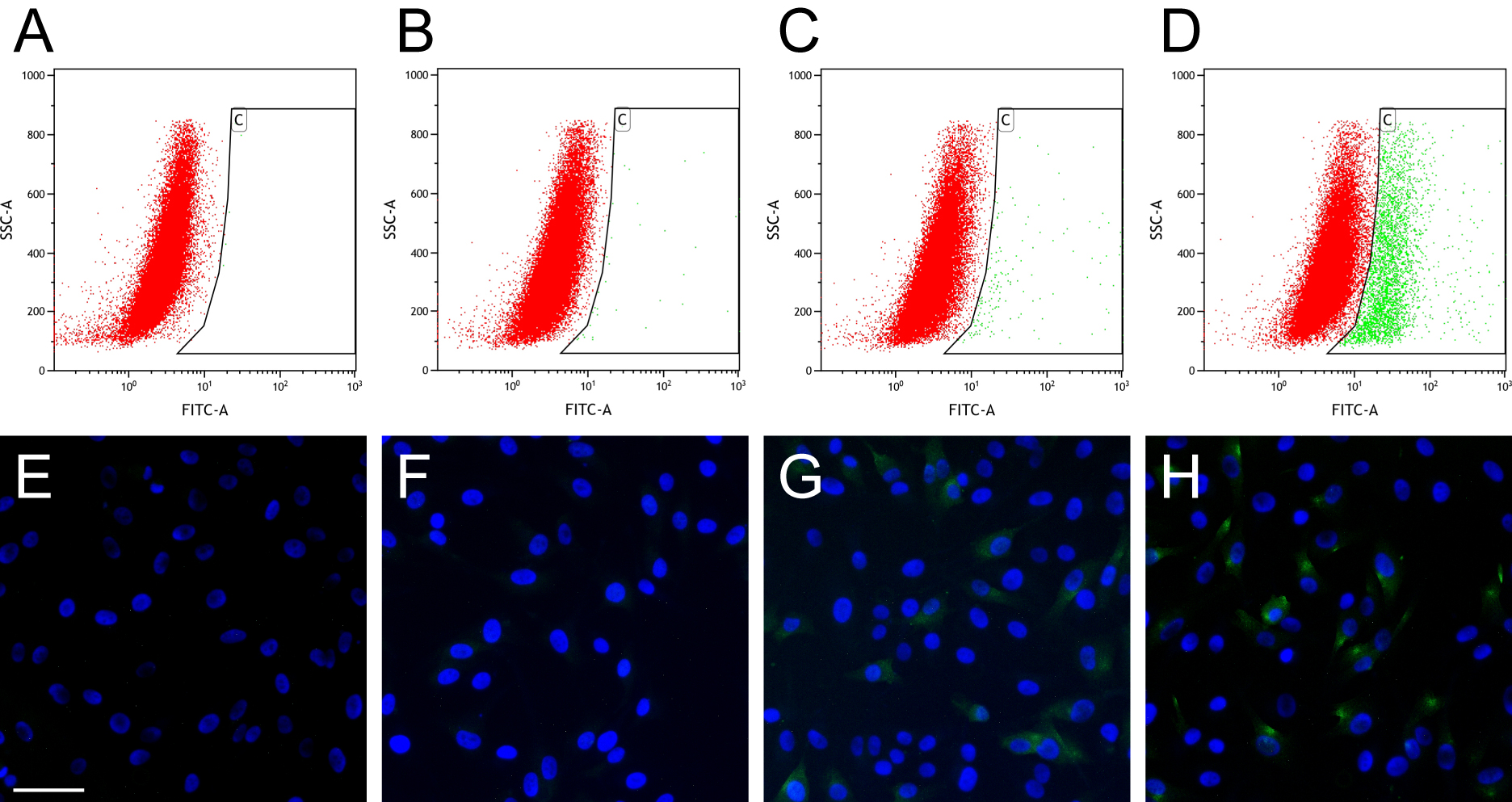

Supplement: Supplementary file 3 — Figure S3. Specificity of m3C2 anti-ABCB5 antibody on SK-MEL-28 cells is proven by FACS and immunofluorescence methods. (A-D) SK-MEL-28 cells were analyzed for surface expression of ABCB5 by incubation of 2.5 × 10^5^ cells for 30 min at 4 °C with m3C2-1D12 anti-ABCB5 antibody or MOPC-31C mouse isotype control antibody (10 μg/ml). This was followed by incubation with FITC-conjugated goat anti-mouse secondary antibody (PharMingen) and single-color flow cytometry. Panels depict cytometry-scatter plots of unstained (A), only secondary-antibody stained (B), isotype plus secondary-antibody stained (C), or anti-ABCB5 plus secondary-antibody stained samples (D). Gate C was used to count ABCB5-positive cells. This contained 0.34% ± 0.15% (mean ± SD) and 6.64% ± 1.46% (mean ± SD) of cells in C and D, respectively. (E-H) Specificity of m3C2-1D12 anti-ABCB5 antibody on immunofluorescence of SK-MEL-28 cells was tested using standard protocols in the presence of FITC-conjugated secondary antibody only (E) or of m3C2-1D12 plus FITC-conjugated secondary antibody (F-H). In addition, primary antibody binding was competed by incubation of 2 μM ABCB5 epitope peptide (F) or scrambled peptide (G). Scale bar: 20 μm. (JPG 962 kb) [file 12885_2019_5606_MOESM3_ESM.jpg]

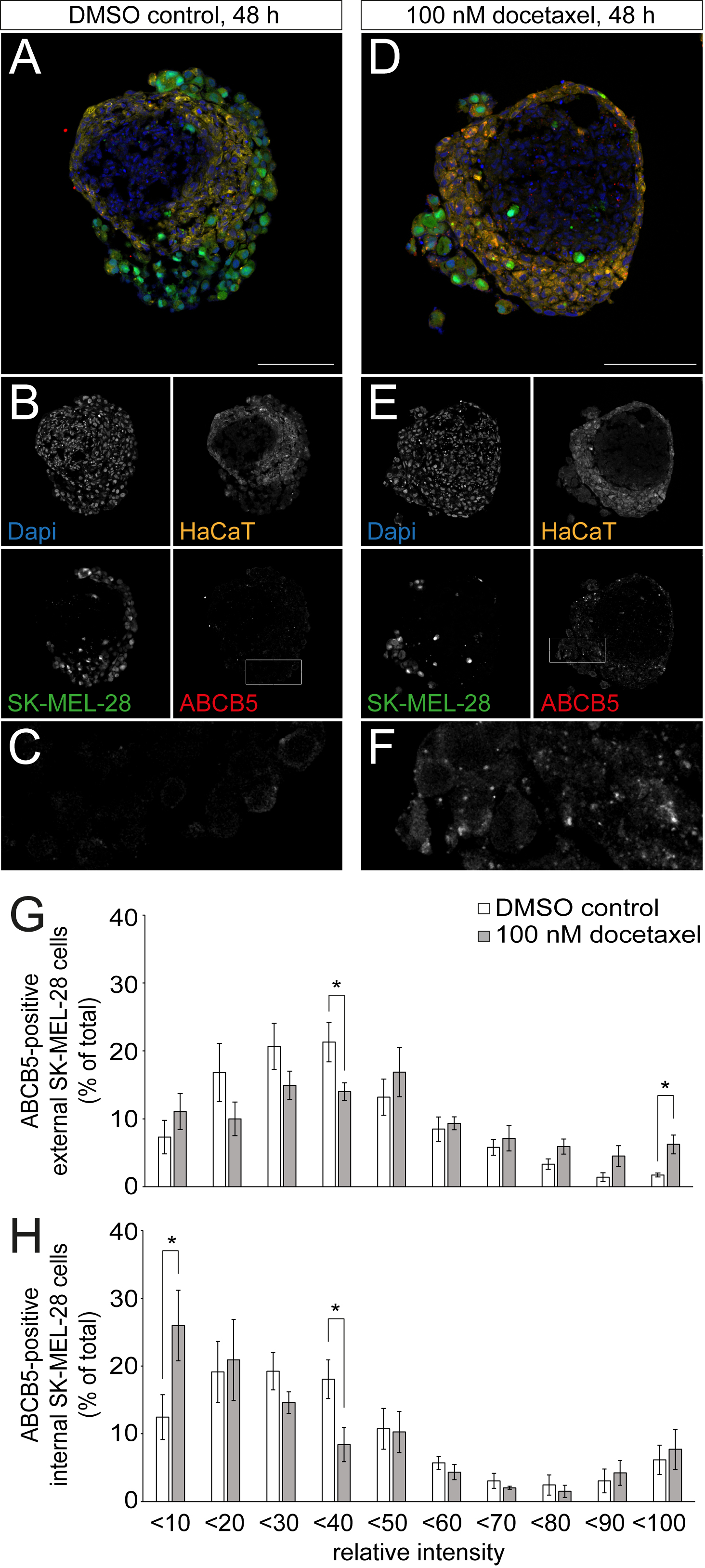

Supplement: Supplementary file 4 — Figure S4. Enhancement of ABCB5-signals in keratinocytes and external melanoma cells upon docetaxel treatment is confirmed by a second anti-ABCB5 antibody. Tri-culture spheroids were generated by 3D cultivation of CCD-1137Sk cells for 3 days, followed by the combined addition of HaCaT and SK-MEL-28 cells. HaCaT and SK-MEL-28 cells were labeled with CellTrackerRed CMPTX and CellTrackerGreen CMFDA dye, respectively. After another 2 days, tri-culture spheroids were treated with 0.01 ‰ of DMSO as control (A-C) or 100 nM docetaxel in DMSO (D-F) for 48 h. Spheroids were cryosectioned into 10-μm thick slices and immunostained with mouse anti-ABCB5 antibody MA5–17026. (A and D) Overlay images of the confocal sections shown in B and E. In overlays, ABCB5 signals, melanoma cells, keratinocytes, and nuclei are depicted in red, green, yellow, and blue, respectively. Scale bars: 100 μm. (C and F) Detail images of ABCB5 signals from boxed regions in B and E. (G-H) Quantification of the relative intensity of ABCB5-positive external (G) and internal (H) SK-MEL-28 cells (percentage of total). Given is mean ± SEM (n = 4 independent experiments; * P < 0.05, ** P < 0.01). For each experiment, ≥ 3 spheroids were analyzed. (JPG 1327 kb) [file 12885_2019_5606_MOESM4_ESM.jpg]

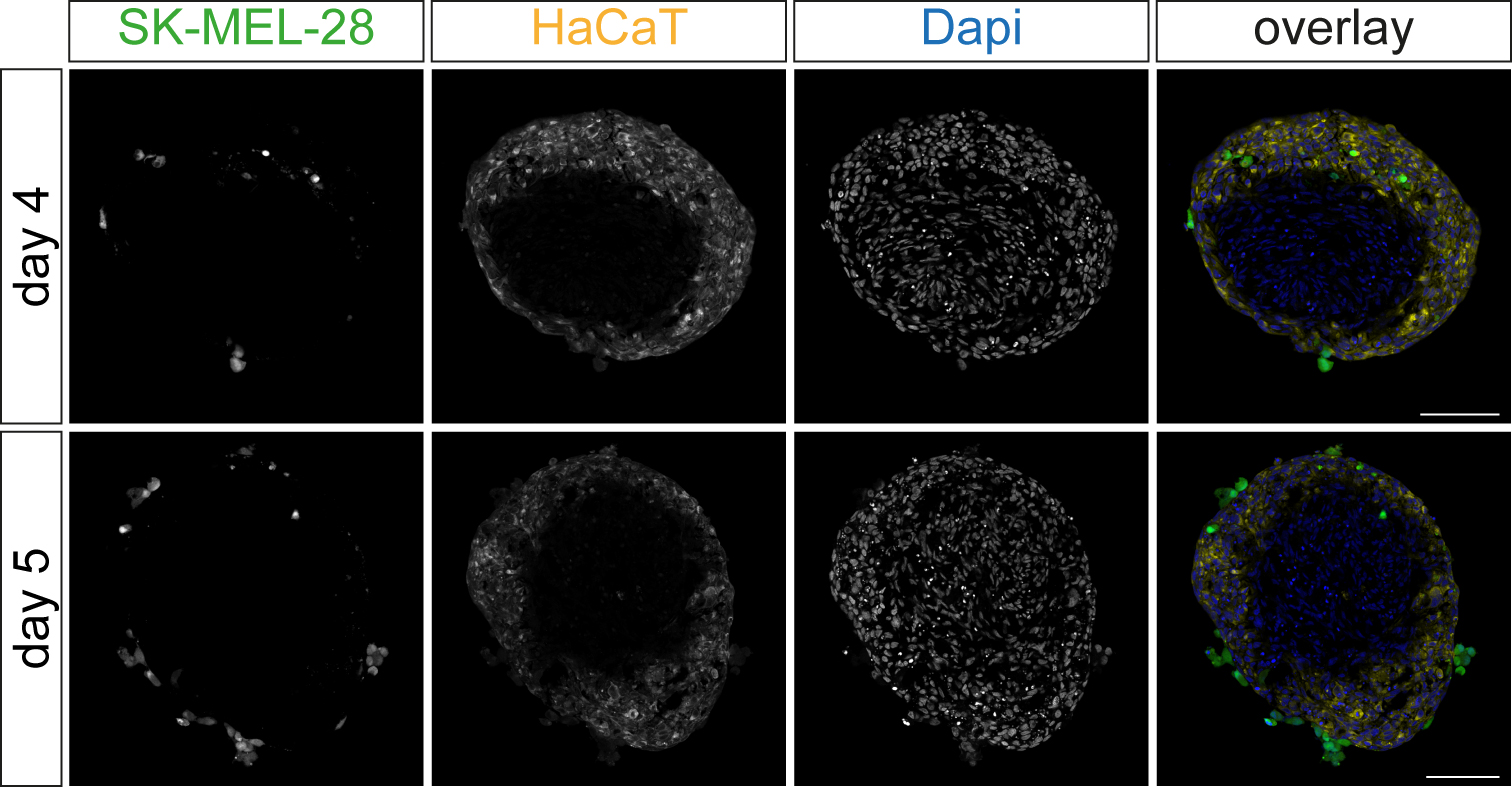

Supplement: Supplementary file 5 — Figure S5. Accumulation of external melanoma cells in tri-cultures is an active separation process. Tri-culture spheroids were generated by 3D cultivation of fibroblasts for 3 days, followed by simultaneous addition of keratinocytes and melanoma cells. HaCaT and SK-MEL-28 cells were pre-labeled with CellTrackerRed CMPTX and CellTrackerGreen CMFDA dyes, respectively. After another one (‘day 4’, upper row) or 2 days (‘day 5’, lower panels), tri-culture spheroids were cryosectioned into 10-μm thick slices and stained with Dapi. Representative confocal images are shown. While most melanoma cells were embedded in the keratinocyte ring on day four, they segregated from keratinocytes on day five and either accumulated in the periphery of the culture (‘external’ melanoma cells) or within the fibroblast core (‘internal’ melanoma cells). The fibroblast core is located in the center of the tri-culture and identified as Dapi-positive plus CellTracker-negative. Scale bars: 100 μm. (JPG 467 kb) [file 12885_2019_5606_MOESM5_ESM.jpg]
